# Pulsed direct current field-induced thermal stability and phase transformation of nanodiamonds to carbon onions

**DOI:** 10.1039/c9ra01022j

**Published:** 2019-05-07

**Authors:** Faming Zhang, Kuowei Fan, Jin Yu, Farhad Saba, Jing Sun

**Affiliations:** Jiangsu Key Laboratory for Advanced Metallic Materials, School of Materials Science and Engineering, Southeast University 211189 Nanjing China fmzhang@seu.edu.cn +86 25 5209 1091 +86 25 5209 1091; State Key Laboratory of High Performance Ceramics and Superfine Microstructure, Shanghai Institute of Ceramics, Chinese Academy of Sciences 200050 Shanghai China

## Abstract

The pulsed DC field-induced thermal stability of nanodiamonds (NDs) and their transformation to carbon onions lack detailed understanding. In this study, a comprehensive study was conducted, analyzing the thermal stability of NDs and the optimum conditions required for the formation of carbon onions, using spark plasma sintering (SPS) utilizing ON–OFF DC pulse energizing. X-ray diffraction, Raman spectroscopy and electron microscopy were employed to monitor the phase transformation. Experimental results showed that NDs could almost remain stable until 950 °C under 60 MPa pressure. As the temperature was increased, amorphous carbon appeared on the surface of NDs, and then, graphitization began. At 1300 °C, lamellar graphite structures were formed and kept stable with increasing holding time, but no carbon onion was found. The optimum parameters for the synthesis of carbon onions from NDs *via* SPS are the temperature of 1400 °C and holding time of 15 minutes under a pressureless condition. The pressureless condition during the SPS processing creates a more favourable environment for the ND graphitization and curling into spherical carbon onions. The existence of pressure during the SPS processing can improve the thermal stability of NDs, delay the initial temperature for the graphitization transition of NDs and inhibit the graphite layer curling to form carbon onions.

## Abstract 2

The pulsed DC field-induced thermal stability of nanodiamonds (NDs) and their transformation to carbon onions lack detailed understanding.

## Graphical abstract

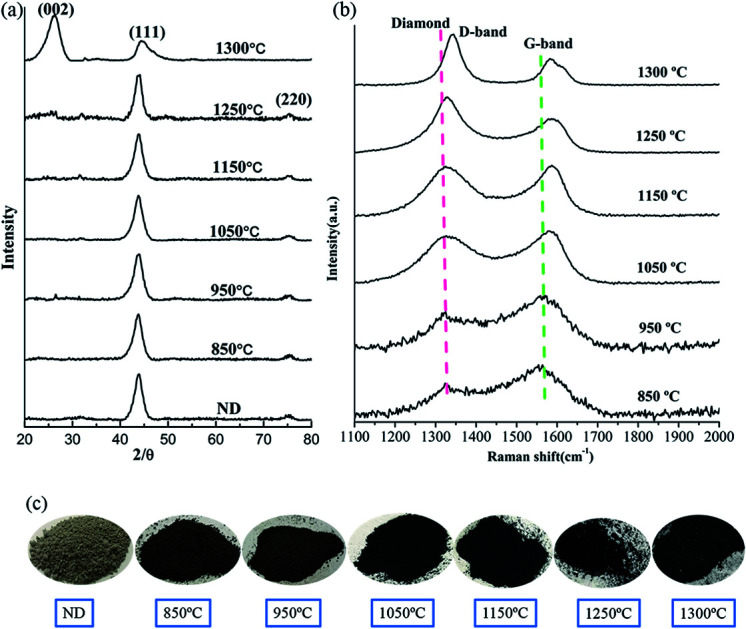

## 1. Introduction

Nanodiamonds (NDs), diamonds with a nanoscale size, were discovered dozens of years ago,^1^ and the investigation of their properties and applications has been ongoing ever since. Similar to their larger counterparts, NDs show many outstanding physical properties including high hardness, Young's modulus, thermal conductivity and electrical resistivity.^2,3^ NDs have been widely used in polymer and metal matrix composites^4–7^ due to their excellent mechanical properties, high surface areas and tunable surface structure.^8,9^ Spark plasma sintering (SPS), also known as field assisted sintering technique (FAST), is an electric field-assisted sintering process utilizing ON–OFF DC pulse energizing.^10^ SPS provides a special physical condition (a pulsed DC field) for studying the stability of a nanomaterial. In view of its rapid heating rate, short sintering time and controllable microstructure, SPS has been increasingly utilized in the preparation of nanocomposite materials in recent years. Therefore, a better understanding of the thermal stability of NDs at various parameters in SPS is useful for a comprehensive understanding of the state of NDs in nanocomposites under different preparation conditions.

Carbon onions, also known as onion-like carbons (OLCs), are a relatively new member of the fullerene family. They were first discovered by Iijima in 1980 (ref. 11) and described by Ugarte in 1992.^12^ Carbon onion has a characteristic structure consisting of 3–8 concentric quasi-spherical and polyhedral-shaped graphitic layers close to one another, and the number of carbon atoms in the second and the third layers increases in terms of 60 *N*^2^ (*N* indicates the number of layers).^13^ Interestingly, the distance between the graphitic layers is 0.335 nm, and it is approximately equal to the distance between two graphitic planes (0.334 nm);^14^ moreover, the inner core is a hollow structure.^15^ Since carbon onions possess unique characteristics as compared to other carbon allotropes (such as diamond, graphene, or carbon nanotubes), they have attracted significant research interest. At present, a variety of methods, for example, electron-beam irradiation of amorphous carbon soot,^16^ thermal annealing of ND,^17,18^ arc-discharge of amorphous carbon soot,^19^ chemical vapor deposition of CH_4_,^20^ and ion implantation of carbon into the Cu, Ni substrate,^21^ have been developed for the production of carbon onions with different sizes (2–50 nm) and shapes.^15^ Note that before the annealed NDs were used to synthesize carbon onions, their study was limited by the low yield or the requirement for complex and expensive processing equipment.^22^ To date, NDs with the size of 5 nm have been widely used as raw materials for the synthesis of carbon onions *via* thermal annealing, and the synthesized carbon onion has a characteristic size close to the size of the raw material and high conversion rate.^23^

Due to their nano-scale size, unique cage structure and carbon atom hybridization (sp^2^) which is different from that of diamond (sp^3^), carbon onions have attracted significant interest for various applications. Potential applications include but are not limited to solid state lubricants,^24^ electrode materials for super-capacitors with high power density,^25^ electromagnetic shielding,^26^ conductive additives,^27^ super-hard diamonds,^28,29^ and composite materials.^5,30,31^ There have been a few studies on the thermal transition of NDs to carbon onions.^14,15^ However, most of them described the effects of the annealing temperature and atmosphere on the shape and the properties of carbon onions in a normal furnace,^32–34^ while the effect of pressure during annealing on the formation of carbon onions was not investigated. At the same time, the pulsed DC field induced phase transformation from ND to carbon onion *via* SPS has not yet been reported in detail. Understanding the transformation characteristics of carbon onions under different production conditions is essential for further understanding of their properties. Therefore, a detailed study regarding the transition of ND in the pulsed DC current field is required.

In the present study, the thermal stability and the phase transformation of NDs under pulsed DC current field and different SPS conditions were investigated in detail. The optimum conversion conditions were also studied for the synthesis of carbon onions using SPS with NDs as raw materials. In order to verify the experimental results, the samples were characterized by X-ray diffraction (XRD), Raman spectroscopy, transmission electron microscopy (TEM), scanning electron microscopy (SEM), laser thermal conductivity (LTC), and thermal analysis (TA). These experiments provide a significant amount of information which can be helpful for understanding the thermal stability of NDs as well as the mechanism of the phase transformation from ND to carbon onion.

## 2. Experimental

Nanodiamonds (NDs) used in this experiment were synthesized by the detonation technique with the average diameter of 5 nm (purity > 98%) and mean specific surface area of 350 m^2^ g^−1^ (Tianjin Qianyu Superhard materials Co. Ltd. China). The ND powders were pressed into a graphite die for SPS treatment to form disk-shaped samples of 20 mm diameter and 3–5 mm thickness. The spark plasma sintering (SPS) system used in this experiment was a Model HP-D5 FCT-SPS (FCT systeme GmbH, Germany), and the applied direct current for SPS was about several thousand A with a pulse duration of 10 ms and an interval of 5 ms. In the first series of experiments, the sintering parameters of the samples were set as follows: an axial pressure of 60 MPa in vacuum, sintering temperature of 850–1300 °C, heating rate of 25 °C min^−1^, holding time of 15 min, and the entire sintering duration of 90 min. In the second part of the study, the sintering parameters were as follows: constant temperature of 1300 °C, pressure of 60 MPa and heating rate of 25 °C min^−1^, which allowed the holding time to vary from 10 to 25 minutes. Finally, to further verify the effect of pressure on the conversion of NDs to carbon onions, the samples sintered at 1200–1400 °C without pressure with holding time of 15 minutes were also examined.

In order to explore the thermal stability of NDs and the mechanism of their conversion to carbon onions, the samples were characterized using an X-ray diffractometer (XRD, D8-discovery, Bruker) with a Cu-Kα monochromatic radiation source and a Raman spectrometer (Thermo Fisher, 532 nm). Transmission electron microscopy (TEM, Tecnai, FEI) with selected area electron diffraction (SAD) was used to observe the microscopic morphology of the samples after sintering. Scanning electron microscopy (SEM, FEI) was used to observe the surface morphology of the sintered samples. Synchronous thermal analyzer (STA449 F3, NETZSCH) was also utilized for thermal analysis of the specimens. Thermal conductivity of the samples was measured using a laser thermal conductivity meter (LFA467, China).

## 3. Results and discussion

### 3.1 Effect of SPS pressure on the thermal stability of NDs

**Fig. 1:**
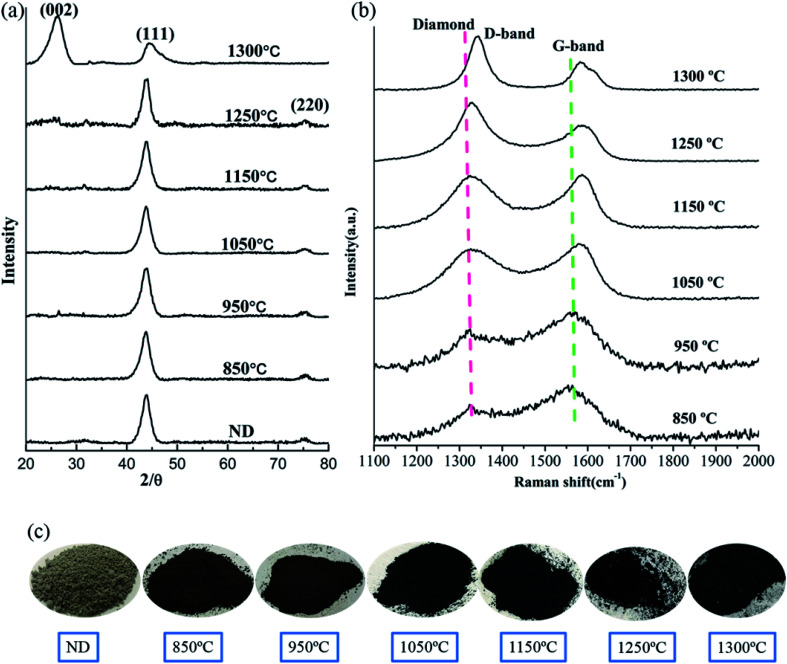
XRD patterns of the raw NDs and the spark plasma-sintered NDs at various temperatures under 60 MPa pressure (a), Raman spectra of the NDs sintered at different temperatures under 60 MPa pressure (b), an image of the raw NDs and the NDs sintered at different temperatures under the SPS pressure of 60 MPa (c).

Fig. 1a demonstrates the XRD patterns of the raw NDs as well as the spark plasma sintered NDs at different temperatures under 60 MPa pressure. It is obvious that two distinct diffraction peaks at 2*θ* = 43.8° and 2*θ* = 76.5° appeared in the XRD patterns of the NDs before and after SPS at different temperatures up to 1250 °C (Fig. 1a). The mentioned peaks can be ascribed to the (111) and (220) reflections of diamond, respectively. It is worth noting that the XRD data of these samples show almost no change in the two characteristic peaks of diamond with increasing temperature, regardless of the position, intensity and shape of the peak. The broadening of the diffraction peaks is related to the size of the raw materials used, compared with the diffraction peaks obtained for the microcrystalline diamond.^22^ However, it can be seen clearly from Fig. 1c that the color of the raw ND powders changes from light gray to dark gray at the intermediate temperature and eventually to black. The change in color can be attributed to graphitization, occuring during sintering of the samples. Therefore, the surface effects are less noticeable and the larger ND crystals contribute more to the overall XRD intensity. The XRD signal from the surface sp^2^ carbon is weak and is overshadowed by the more intense scattering of the diamond phase, which is formed at the onset of the transformation process. For small ND crystals, which were fully converted to sp^2^ carbon at lower temperatures and produced nonplanar graphitic carbon, their (002) peak would be very weak if detectable at all and easily be overshadowed by the strong signal of larger ND crystals that possess little or no sp^2^ carbon.^35^ In addition, using the Debye–Scherrer equation, the average size of the particles in all samples was calculated to be 5 nm around. Accordingly, based on this analysis, the grain size of the samples did not change significantly before and after SPS. On the other hand, the absence of the graphite (002) peak in the XRD results of the sintered samples below 1250 °C can be explained by the formation of very thin amorphous carbon or sp^2^ graphite shells on the surface of the ND particles. Therefore, it can be inferred that the ND could maintain stability under pressure below 1250 °C. At 1300 °C, the XRD pattern of the sample contains the (002) graphite peak near 26° (Fig. 1a) due to graphitization of NDs at higher temperature. The experimental results demonstrate that the graphitization transitional temperature of ND can be delayed and the stability of NDs can be enhanced by appropriate pressure in the SPS.

The Raman spectroscopy results of the corresponding samples after SPS are presented in Fig. 1b. The red dotted (∼1332 cm^−1^) line represents the diamond peak, and the green dotted line (∼1580 cm^−1^) corresponds to the G-band. The presence of the diamond peaks in the sintered samples (below 1250 °C) is also consistent with the XRD results (Fig. 1a). As the temperature increases, the intensity of the G-band decreases and shifts to larger wavenumbers. Since Raman spectroscopy measurements are sensitive to the onset of sp^2^ carbon formation at the crystal surface, a slight transition from sp^3^ to sp^2^ on the surface of the samples can be observed. It can be speculated that the formation of the thin amorphous carbon layer or the graphite shells (localized sp^3^–sp^2^ transformation) on the surface of ND (NDs are coating the inner layer) weakens the corresponding Raman signal and explains the change in the G-band.^3^ Similar to the temperature dependence of several important intensity ratios that are typically used to evaluate the composition and the structural ordering of carbon materials, the intensity ratio *I*_Dia_/*I*_G_ can be used to evaluate the sp^3^/sp^2^ ratio or the diamond content.^36^ The corresponding results are presented in Table 1. As the temperature increases, it can be found that the ratio of *I*_Dia_/*I*_G_ increases gradually. This result also indirectly supports the transformation of sp^3^ to sp^2^ on the surface of ND and slight graphitization. The presence of the D-band (1343 cm^−1^) at 1300 °C and the corresponding XRD results in Fig. 1a prove that under these conditions most of NDs will be transformed into the graphite-like structures.

**Table 1:** Raman peak characteristics of the spark plasma-sintered NDs at various temperatures under 60 MPa

NDs	Pos. (cm^−1^), diamond	Pos. (cm^−1^), G	Int. (a.u), diamond	Int. (a.u), G	*I*_Dia_/*I*_G_
850 °C	1326	1566	268	440	0.61
950 °C	1326	1566	301	439	0.69
1050 °C	1326	1580	328	367	0.89
1150 °C	1326	1588	450	472	0.95
1250 °C	1326	1590	267	177	1.56
1300 °C	1343(D)	1593	195(D)	104	1.87(*I*_D_/*I*_G_)

**Fig. 2:**
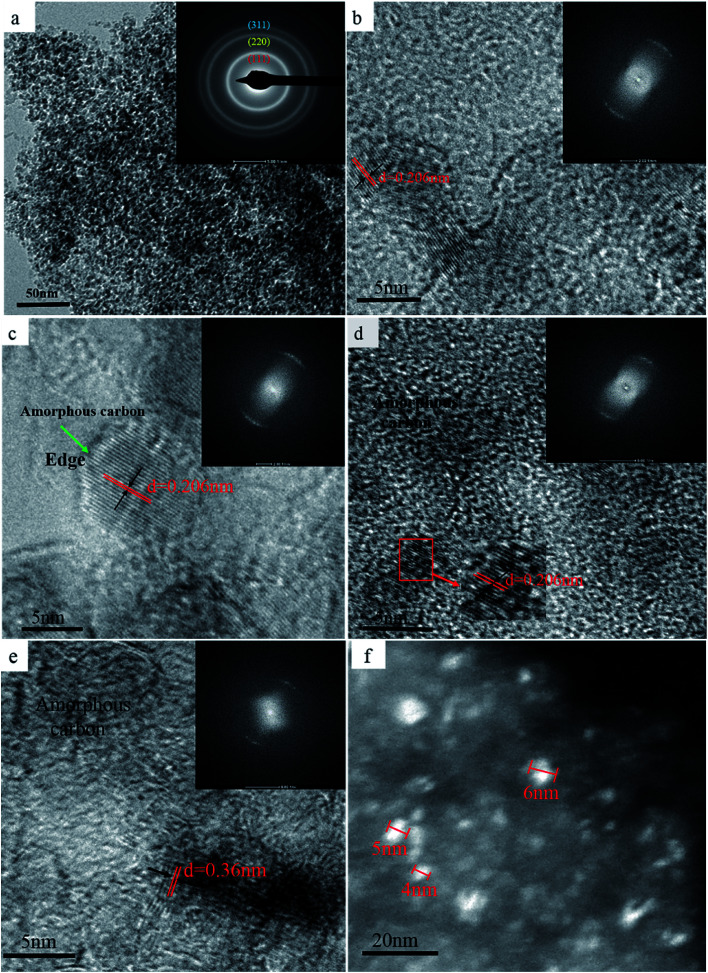
TEM bright-field images of the NDs after SPS processing under 60 MPa and at 850 °C (a), 950 °C (b), 1050 °C (c), 1150 °C (d), and 1250 °C (e) and (f) the dark field image.

In order to confirm the above results, the morphology of each sample after SPS was evaluated using TEM, and the results are shown in Fig. 2. The ND raw materials after sintering at 850 °C did not exhibit any noticeable structural changes, which is confirmed by the presence of three noticeable diamond diffraction rings in the diffraction pattern (inset) of Fig. 2a. Furthermore, the 950 °C processed sample data showed that the spacing of the lattice fringes with *d* of 0.206 nm corresponds to the (111) crystal plane of diamond (Fig. 2b). At 1050 °C, thin amorphous carbon begins to appear at the edges of the NDs grains, but most of the grains still retain the original structural features (Fig. 2c). As the temperature continues to rise, amorphous carbon is formed at the edges of the whole ND grains, while the NDs with stable structure are coated with amorphous carbon (Fig. 2d). The broadening of the diffraction ring in Fig. 2d also confirms the formation of amorphous carbon in the sample after sintering, as compared to the diffraction ring in Fig. 2b. It is noteworthy that graphite fragments with about three layers appear locally in the sintered samples when the temperature rises to 1250 °C. At this time, most of the samples are composed of amorphous carbon and untransformed diamond. Interestingly, the layer spacing of the graphite fragments is approximately 0.36 nm, which is close to that of the perfect graphite sheets (0.34 nm). It can be concluded that the sp^3^ to sp^2^ transformation occurs locally in the sample. In addition, the results shown in Fig. 2d and e confirm the validity of the discussion regarding the D-band transfer in the above Raman spectroscopy data. Fig. 2f shows the dark field TEM image of the sample after sintering at 1250 °C. It was determined that the crystallite size of the sample was around 5 nm, which is consistent with the crystallite size, calculated using the Debye–Scherrer equation based on the XRD patterns of Fig. 1a. Therefore, it can be concluded that the appropriate pressure applied in SPS does not cause coarsening of the NDs crystal grains. Moreover, no extensive graphitization of the samples was detected, which proves that pressure can improve the thermal stability of NDs in SPS to a certain extent.

### 3.2 Phase transformation from NDs to carbon onions

Normally, when a certain annealing temperature is reached, carbon atoms acquire sufficient energy allowing the ND to carbon onion transformation to occur without a catalyst. However, at the beginning of the transformation process, graphite fragments are formed on the surface of the ND grains.^22^ Based on the results of the thermal stability analysis of ND in the previous section (3.1), the effect of holding time on the transformation of NDs to carbon onions under the appropriate SPS pressure was investigated at the constant temperature of 1300 °C.

**Fig. 3:**
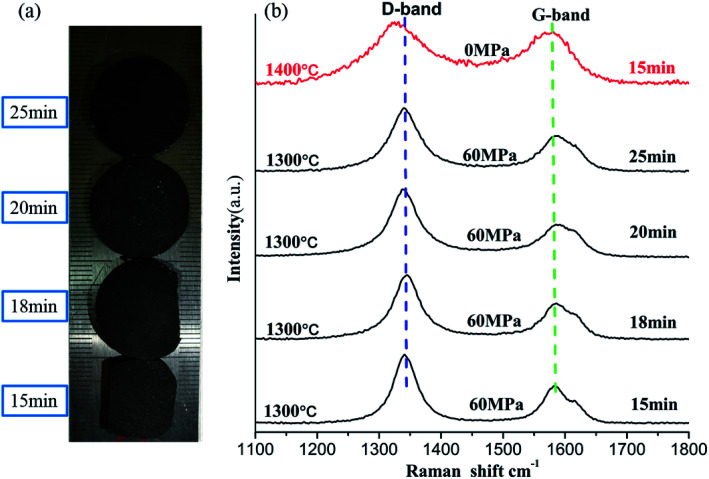
Image (a) and Raman spectra (b) of the NDs spark plasma sintered at 1300 °C and 60 MPa for different holding times; the red line in (b) represents the Raman spectrum of the NDs annealed at 1400 °C without pressure.

Fig. 3a shows the photographs of the sintered NDs taken at different holding times. It can be seen that the color of the samples has not changed significantly, but it showed obvious densification. Since the sintered samples have a regular shape, the densities can be estimated roughly by using their mass and volume values. In addition, the density measurements and the Raman results are provided in Table 2. Comparing the density results with those of diamond (3.47 g cm^−3^) and graphite (2.25 g cm^−3^), it can be confirmed that the pore structures remained in the bulk samples and the porosities are about 50% or more. Fig. 3b shows the Raman spectra of the samples with different holding times. In Fig. 3b, the blue dotted line near 1343 cm^−1^ indicates the D-band, while the green dotted line near 1585 cm^−1^ indicates the G-band. The D-band originates from the defects and disorder of the sp^2^-hybridized carbon, and the G band comes from stretching of the C–C bonds in the sp^2^-hybridized carbon materials, which can also be used to indicate the degree of graphitization.^37,38^ As the holding time is increased, there is no significant difference in the location and shape of the D-bands and the G-bands in Raman spectra of the samples (at 1300 °C). Since planar graphite is the most stable structure for particles above 5–10 nm in diameter,^39^ we speculate that the carbon atoms in the sintered samples exist in the form of planar graphite. Simultaneously, the D-to-G band ratio (*I*_D_/*I*_G_) provides information on the structural ordering of the sp^2^-phase (*e.g.*, level of graphitization).^40^Table 2 summarize the peak positions and the intensity ratios (*I*_D_/*I*_G_) for all sintered samples. It is worth noting that the ratio of *I*_D_/*I*_G_ did not show a significant change with increasing holding time. Thus, the prolongation of holding time under pressure does not significantly change the structure of carbon atoms in the sample. Instead, the carbon atoms should be stable in the sample in a certain structural form of the sp^2^ hybridized carbon.

**Table 2:** Densities and Raman results of the spark plasma sintered NDs

NDs	Density (g cm^−3^)	Pos. (cm^−1^), D	Pos. (cm^−1^), G	Int. (a.u) D	Int.(a.u), G	*I*_D_/*I*_G_
1300 °C–15 min	0.86	1344	1584	150	77	1.82
1300 °C–18 min	0.81	1344	1583	129	72	1.80
1300 °C–20 min	0.83	1343	1586	156	79	1.93
1300 °C–25 min	0.90	1341	1587	121	68	1.76

**Fig. 4:**
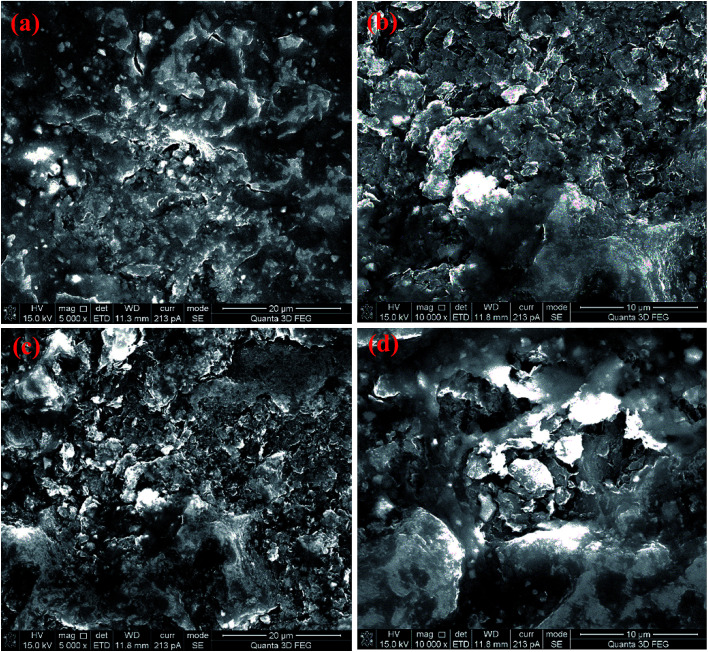
SEM images of the NDs spark plasma sintered at 1300 °C and 60 MPa with different holding times of 15 min (a), 18 min (b), 20 min (c) and 25 min (d).

The SEM micrographs of the samples with different holding time of 15, 18, 20 and 25 min at 1300 °C and 60 MPa are provided in Fig. 4a–d, respectively. Under the pressure of 60 MPa, the sintered samples retain a large number of lamellar pore structures, which do not change significantly with the prolongation of holding time. Therefore, it can be concluded that applying a certain amount of pressure in SPS is not only conducive to the consolidation of powder samples, but also can be combined with other parameters to synthesize porous materials.

**Fig. 5:**
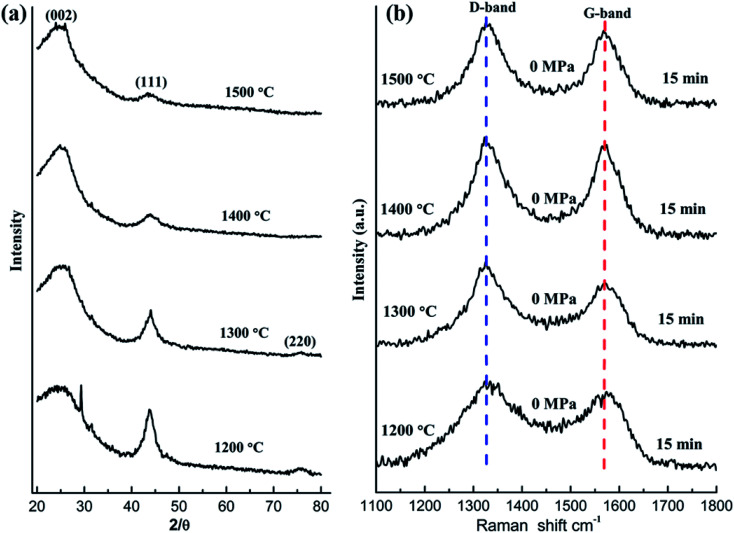
XRD patterns (a) and Raman spectra (b) of the NDs annealed at different temperatures without pressure.

Fig. 5a displays the XRD results of the samples sintered at different temperatures in the absence of pressure. All samples exhibit a characteristic peak at 2*θ* = 44°, which originates from the (111) planes of diamond (sp^3^), and the (002) planes of graphitic carbon (sp^2^) appear at 2*θ* = 26°. As can be seen in Fig. 5a, the intensity of the (111) peaks, which indicate the presence of diamond in the sample, decreases gradually with increasing temperature while the shape tends to broaden. Since the broadening of the diffraction peaks can be used to evaluate the change in the diamond grain size of the sintered samples,^41^ we speculate that the graphitization transformation rate of diamond is related to the size of the raw ND with an increase in temperature. Considering the size difference of raw NDs, larger ND crystals appear only partially converted due to slower annealing kinetics.^35^ There are still a few residual ND grains in the samples even at 1500 °C. Moreover, compared with the results shown in Fig. 1a, the results shown in Fig. 5a further support that the existence of a certain pressure can increase the graphitization transition temperature of ND.

The Raman spectra of all sintered samples show the D- and G-mode characteristic peaks between 1200 and 1700 cm^−1^ (Fig. 5b). With the increase of temperature, the position of the D- and G-bands did not shift significantly in the Raman spectra of the sintered samples, but below 1300 °C, the D- and G-bands showed asymmetry following widening. Based on the analysis of other studies,^32,33^ this phenomenon can be ascribed to the disorder of graphite structure in the samples. That is to say, at relatively low temperatures, a thin layer of the sp^2^-hybridized carbon is formed that at higher temperatures encompasses all ND particles. Furthermore, similar to what has been reported in other studies,^32,42–45^ the *I*_D_/*I*_G_ ratio of carbon onions synthesized at low temperatures (∼1000 °C) is rather low (∼1.6); subsequently, at temperatures above 1100 °C, the *I*_D_/*I*_G_ ratio decreases constantly with the increasing annealing temperature. From the results in Table 3, it can be seen that the *I*_D_/*I*_G_ ratio decreases gradually to around 1 (∼1400 °C) with the increasing temperature. Therefore, we speculate that the long-range ordering of graphitic carbon will constantly be enhanced at around 1400 °C.

**Table 3:** Raman peak characteristics of the spark plasma-sintered NDs

NDs	Pos. (cm^−1^), D	Pos. (cm^−1^), G	Int. (a.u), D	Int. (a.u), G	*I*_Dia_/*I*_G_
1200 °C–15 min	1329	1572	32	28	1.14
1300 °C–15 min	1328	1574	43	33	1.30
1400 °C–15 min	1326	1570	40	38	1.05
1500 °C–15 min	1325	1572	45	41	1.09

**Fig. 6:**
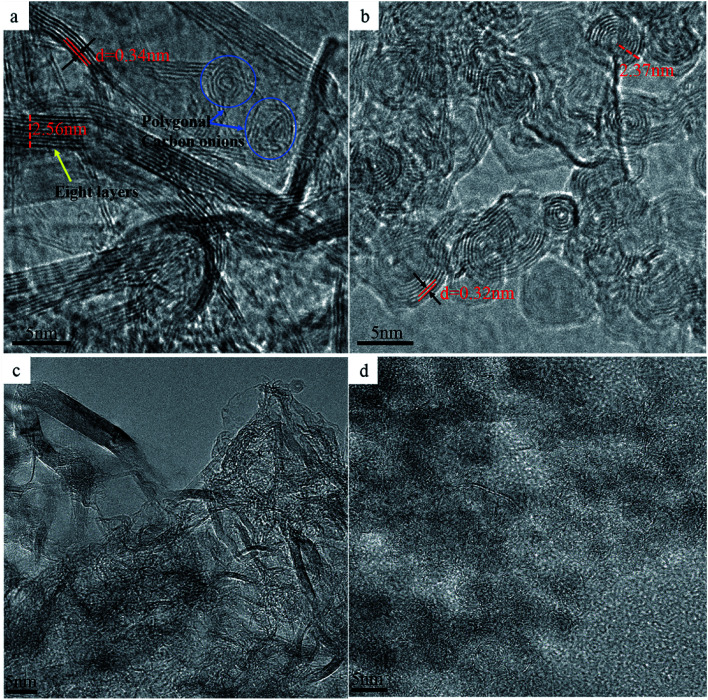
TEM images of the NDs spark plasma sintered at 1300 °C and 60 MPa for 15 minutes (a) and at 1400 °C without pressure for 15 minutes (b), spark plasma sintered at 1500 °C for 15 min at 9.55 MPa (c) and without pressure (d).

To confirm the above analysis, high resolution TEM (HRTEM) images of the selected samples with different sintering parameters were obtained (Fig. 6). As can be seen in Fig. 6a, most of the NDs in the sintered samples are transformed into graphite sheets (*d* = 0.34 nm) with 6–8 layers and average thickness of 2.56 nm, which is in agreement with Raman spectroscopy data (Fig. 3b). Unlike the thermal annealing processes of ND reported in the literature, graphitization occurs from the outer layer to the inner layer on the surface of NDs, eventually yielding carbon onions.^32,34,46^ Our experimental results show that even above the transition temperature at which NDs transform to carbon onions, pressure is beneficial for maintaining the graphitization structure of carbon atoms and effectively inhibiting their conversion to carbon onions. Nevertheless, polygonal carbon onions were also found in the local regions of the sintered samples with approximately 3–4 hollow layers. For this reason, the ND particles located near the inner wall of the graphite mold and those located in the middle of the mold will be subjected to different sintering conditions. To illustrate the rationality of this explanation, the HRTEM image of the sample produced at 1400 °C in the absence of pressure is presented in Fig. 6b. It can be seen that under these conditions, NDs were almost completely converted to spherical carbon onions containing approximately 6–8 graphitic layers with the layer spacing of 0.32 nm and the average radius of 2.37 nm. Comparing the TEM images in Fig. 6a and b, although there is no direct evidence that the multi-layer graphite sheets are bent and connected to carbon onions at the same time, the intermediate process of graphitization of NDs to carbon onions can be confirmed. Furthermore, once the temperature reaches a certain value, the pressure does not interfere with graphitization of NDs, but it can effectively suppress the bending and curling of the graphite sheet layers and help maintain a large radius of curvature, showing a strip-like structure in which the layers are parallel to each other. Under these conditions, strip graphite sheets are more thermodynamically favorable than carbon onions. Therefore, we conclude that the transformation of NDs to carbon onions requires an intermediate process of graphitization under the low or no pressure conditions. Fig. 6c shows the TEM image of the NDs sintered at the higher SPS temperature of 1500 °C and at very low pressure of 9.55 MPa (3 kN) for 15 min holding time. However, the spherical or quasi-spherical structured carbon onions have not been detected. The final products, as shown in Fig. 6c, are the irregular layered graphite. At the same temperature and holding time in the absence of pressure, spherical and quasi-spherical shaped carbon onions can be obtained (Fig. 6d). As compared to the results of 1400 °C (Fig. 6b), it can be seen that the microstructures of carbon onions got worse by increasing the temperature to 1500 °C. The optimum parameters for synthesizing carbon onions from NDs *via* SPS are the temperature of 1400 °C and the holding time of 15 minutes in the absence of pressure. It has been confirmed that applying pressure during SPS cannot induce the formation of spherical or quasi-spherical structured carbon onions. The pressure inhibits the graphite layers curling into carbon onions. SPS is a fast sintering process, but we chose a low heating rate of 25 °C min^−1^. We have sintered some samples at the heating rate of 50–100 °C min^−1^ in the beginning of the investigation but found that a faster heating rate can lead to the construction of an incomplete carbon onion microstructure. Therefore, the phase transformation from NDs to carbon onions needs a certain amount of time for the complete conversion. Lower heating rate during SPS is suitable for the carbon onion synthesis. Thus, the lower heating rate of 25 °C min^−1^ was selected for studying the effect of temperature and pressure. Most importantly, different from other methods of producing carbon onions which have many shortcomings, such as high energy consumption, long production cycle and low conversion rate, the applied method of synthesizing carbon onions with a regular shape and high conversion rate from NDs *via* SPS under appropriate temperature, short holding time and the absence of pressure will have absolute advantages.

**Fig. 7:**
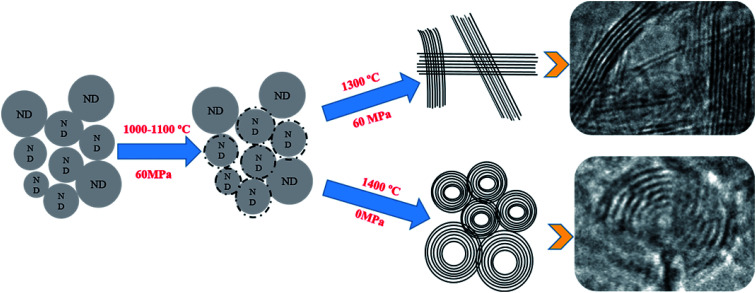
Schematic of the ND structural changes under pulsed DC field with pressure and without pressure.

Based on the results provided in the previous sections (3.1 and 3.2), the schematic illustration of the structural changes in NDs is shown in Fig. 7. The following steps are believed to take place during the annealing process. First, NDs can almost maintain their original structure under pressure when the temperature does not exceed 1000 °C, and only on the surface of the small particles amorphous carbon can be made. Second, at constant pressure, the graphitization process of NDs gradually begins when the temperature continues to rise until all of the carbon atoms in NDs are in the form of lamellar graphite at 1300 °C. On the other hand, the lamellar graphite structure did not change with the increasing holding time, and onion carbon could not be found as a spherical structure. Finally, in the absence of pressure, when the temperature reaches 1400 °C, NDs are converted to carbon onions with regular spherical structures. The conversion to carbon onions from NDs *via* SPS is accomplished at 1400 °C and 15 min holding time in the absence of pressure. The temperature is lower and the time is shorter than those of the other synthetic methods using a normal tube-furnace^34^ and a normal vacuum furnace.^25,33^ It is indicated that plasma, generated during SPS, played the key role and provided most of the energy needed for the carbon onion formation. The momentary pulsed plasma provided energy equivalent to several thousand degree to help the NDs to transform into the carbon onion phase at relatively lower sintering temperatures and shorter time. Thus, this study provided important evidence for the existence of plasma during SPS with no applied pressure.

### 3.3 Thermal analysis of the carbon onion bulk blocks

**Fig. 8:**
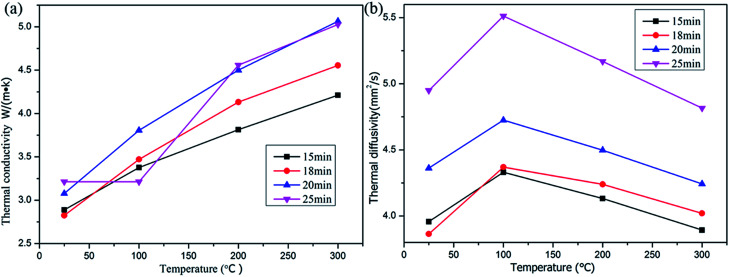
Thermal conductivity (a) and thermal diffusivity (b) of the NDs after spark plasma sintering at 1300 °C and 60 MPa for different holding times.

For the sample shown in Fig. 3a, a thermal conductivity analysis was conducted, and the results of thermal conductivity and diffusivity of the carbon onion bulk blocks are provided in Fig. 8a and b, respectively. Based on Fig. 6a, it can be concluded that the carbon atoms in the sample were almost in the form of layered graphite. Comparing the results of Fig. 8a with the theoretical thermal conductivity of graphite (129 W m^−1^ K^−1^), it is obvious that thermal conductivity of the sintered carbon onion bulk blocks was strongly decreased. The reason for this phenomenon may be the existence of pores (Fig. 4a–d) which reduce the thermal conductivity of the sample. On the other hand, the asymmetry of microstructures and the composition of different phases (gas and solid) at the boundary of the sample matrix inhibit the heat transfer. Interestingly, the thermal conductivity of the samples did not change significantly with the increase of temperature, and the holding time had only a slight effect on the thermal conductivity of the samples (Fig. 8a). Combined with the density variation of the samples in Table 2, the above analysis indirectly demonstrates the stability of this porous structure (Fig. 4a–d). Moreover, according to the thermal diffusion coefficient equation,1
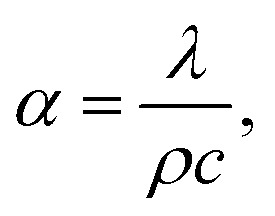
where *α* is thermal diffusivity, *λ* is thermal conductivity, *ρ* is density, and *c* is heat capacity, the thermal diffusivity of the corresponding sample calculated from thermal conductivities (Fig. 8a) as well as densities (see Table 2); the results are shown in Fig. 8b. Since *α* is the indicator of the ability of a material to change temperature, the smaller the value, the more the temperature increase is hindered.

**Fig. 9:**
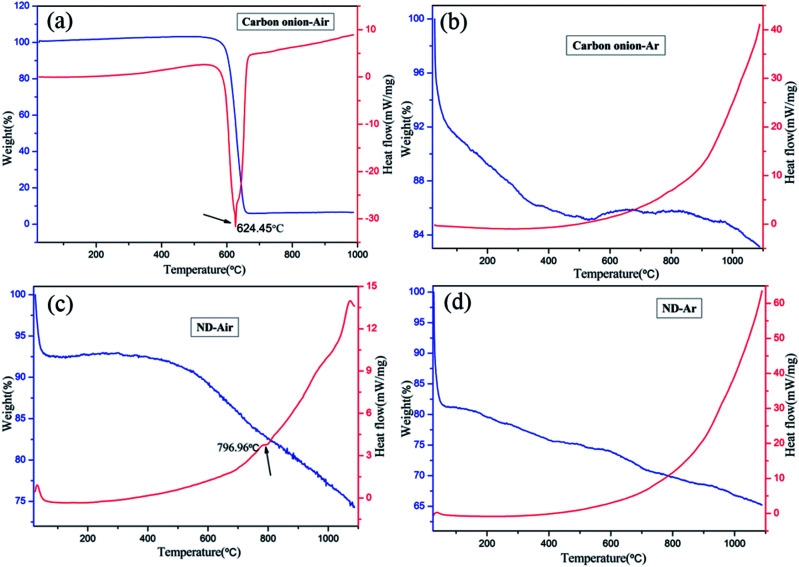
TG and DSC curves of carbon onion prepared *via* SPS at 1400 °C (a and b) and ND (c and d) in air and under an Ar atmosphere measured by a synchronous thermal analyzer at the heating rate of 10 °C min^−1^.

Oxidation of the carbon onion powders, synthesized in this experiment, was analyzed by TG and DSC in air and Ar atmosphere, respectively, and the results are provided in Fig. 9a and b. For comparison, the corresponding results for NDs under the same conditions are also shown in Fig. 9c and d. When comparing the results of Fig. 9a and c, it can be found that carbon onion, formed after the removal of the surface functional groups, shows a higher oxidation resistance than ND. Regarding the TG results of carbon onions in air (Fig. 9a), the maximum weight loss occurs at about 625 °C, corresponding to carbon onion oxidation. The presence of an exothermic peak around 624.5 °C in the DSC curves of the sample supports this result. At the same time, the TG results of ND and carbon onion in Ar atmosphere (Fig. 9b and d) reveal that carbon onion exhibits a lower weight loss rate than ND with increasing temperature. This observation can also be explained by the fact that carbon onions formed at high annealing temperature have more structural ordering of the sp^2^ phase than NDs.

## 4. Conclusion

Spark plasma sintering (SPS) was used to investigate the thermal stability of NDs under different pressure conditions. Under the pressure of 60 MPa, NDs were stable below 950 °C, and only the amorphous carbon appeared at the edges of the local particles. When the temperature exceeded 1200 °C, ND could no longer maintain its structural stability, and the microstructures mostly contained amorphous carbon and graphite fragments. For the samples sintered at 1300 °C and 60 MPa, the formation of carbon onion was not observed with prolonging the holding time, and the carbon atoms existed in the form of regular layered graphite. This structure created a porous matrix structure, showing a significant decrease in the density and thermal conductivity of the sample after sintering.

Furthermore, the optimum parameters for synthesizing carbon onions from NDs *via* SPS were found to be the temperature of 1400 °C and holding time of 15 minutes in the absence of pressure. Finally, we conclude that applying pressure can improve the thermal stability of NDs, delay the initial temperature of the graphitization transition of NDs and inhibit the graphite layer curling to form carbon onions. This important result should be very helpful for future research in the field of carbon onions synthesis and composite materials containing nanodiamond additives produced by SPS. It have great advantages for the fabrication of carbon onions using the SPS under appropriate temperature and short holding time at pressureless condition. In addition, this study can open up general ways for the processing of similar composites with a variety of nano-reinforcements in different shapes and structures.

## Conflicts of interest

There are no conflicts to declare.

## Supplementary Material
